# Insecticide resistance of Dengue vectors in South East Asia: a systematic review

**DOI:** 10.4314/ahs.v21i3.21

**Published:** 2021-09

**Authors:** Mohd Rohaizat Hassan, Noor Atika Azit, Suhaiza Mohd Fadzil, Siti Rasidah Abd Ghani, Norfazilah Ahmad, Azmawati Mohammed Nawi

**Affiliations:** Department of Community Health, Faculty of Medicine, Universiti Kebangsaan Malaysia, Jalan Yaacob Latiff, 56000 Cheras, Kuala Lumpur, Malaysia

**Keywords:** Insecticide resistance, vector management, Aedes, pyrethroid, mortality

## Abstract

**Background:**

The insecticides used widely has led to resistance in the vector and impose a challenge to vector control operation.

**Objectives:**

This review aims to analyse the distribution of insecticide resistance of dengue vectors in South East Asia and to describe the mechanism of insecticide resistance.

**Methods:**

Literature search for articles published on 2015 to 2019 from PubMed, Scopus and ProQuest was performed. Total of 37 studies included in the final review from the initial 420 studies.

**Results:**

Pyrethroid resistance was concentrated on the west coast of Peninsular Malaysia and Northern Thailand and scattered at Java Island, Indonesia while organophosphate resistance was seen across the Java Island (Indonesia), West Sumatera and North Peninsular Malaysia. Organochlorine resistance was seen in Sabah, Malaysia and scattered distribution in Nusa Tenggara, Indonesia. V1016G, S989P, F1269C gene mutation in Aedes Aegypti were associated with Pyrethroid resistance in Singapore and Indonesia. In Malaysia, over-expressed with monooxygenase P450 genes (CYP9J27, CYP6CB1, CYP9J26 and CYP9M4) Glutathione S-transferases, carboxylesterases commonly associated with pyrethroids resistance in Aedes Aegypti and CYP612 overexpressed in Aedes Albopictus. The genetic mutation in A302S in Aedes Albopictus was associated with organochlorine resistance in Malaysia.

**Conclusions:**

Rotation of insecticide, integration with synergist and routine assessment of resistance profile are recommended strategies in insecticide resistance management.

## Introduction

Aedes mosquito is an essential vector for many vector-borne infections. Aedes mosquito species mainly Aedes aegypti is the primary vector, and Aedes albopictus is the vector responsible for transmitting the dengue virus between people. There is four known dengue virus serotype circulating in Asia, Africa and America designated as DENV-1, DENV-2, DENV-3 and DENV-4 transmitted by the Aedes mosquito^1^. Infection to one serotype will produce lifelong immunity to that serotype virus and cross-protection to other serotypes for a few months^1^. Secondary infection with other serotypes will lead to severe infection^1^.

Aedes mosquito species found between latitude 45° North and 35° South in tropics and subtropical countries which closely related to human habitat in urban area^2^. It breeds mostly in a man-made container such as used tyres, plastic container, tin container, and even a small cup can attract the female Aedes mosquito to breed. Aedes mosquito takes about 8 to 10 days to complete the life cycle from eggs to adult^3^. Female adult Aedes mosquito has a strong affinity for human blood and prefers to bite human during dusk and dawn period. Due to its limited flying capacity, it is found mostly inside or outside home or buildings close to human 3.

In 1998 Aedes mosquito was found throughout all continents including North America and Europe, and as the result of expanding geographical distribution, half of the world's population are at risk of dengue infection^4^. Before the 1970s only nine countries reporting severe dengue epidemics, however since the last five decades dengue fever is endemic in more than 125 countries with the western Pacific region, South-East Asia and America are the most affected countries^5^. It estimated that about 390 million dengue infection occur every year throughout the world with 20 000 dengue-related death that is resulting in dengue considered as worsening public health problem^5^. Most countries in South East Asia has a high burden of dengue infection with 3 to 5 years cyclical episodes of epidemics 6. In 2010, 354 009 cases from WHO Western Pacific Region of South East Asian countries which include Cambodia, Lao, Malaysia, Singapore, Philippines and Vietnam with 1075 deaths were reported to World Health Organization (WHO)^6^,^7^. Together with other South-East Asian countries, it contributes to 75% of disease burden^6^.

Environmental change, urbanisation, increase population density, travel and trade activities that allowing movement of the vectors and virus via modern transportation, the emergence of these viruses from their sylvatic reservoirs, has been recognised to contribute to the emergence and re-emergence of dengue in South Asia^8^. Aedes mosquito has a high vectorial capacity and undergone adaptive changes to achieve longevity and to survive in cooler climates^2^. Unplanned urbanisation and weak environmental management cause increase in potential breeding site for Aedes mosquito. High-density population in the capital city and travel activity further increased the dengue epidemic in South Asian countries.

Till date, there is no specific treatment for dengue infection and Dengvaxia vaccine is not widely used^9^. Source reduction, clean-up campaign and larviciding have been promoted widely; however, it was not very successful. Prevention and control of Aedes nowadays are based mainly on integrated vector management which includes integrated use of insecticide by space spraying to reduce the adult mosquito density, larviciding and environmental management^10^. Space-spraying using aerial and truck-mounted ultra-low volume (ULV) is now not recommended because of the low impact on mosquito density reduction and less cost-effective to implement it as a routine intervention 11. There are four significant insecticides commonly used, namely pyrethroid, organophosphate, carbamate and organochlorine. Long term and extensively used of these insecticides in dengue control has led to the development of resistance in the vector, and this will impose a challenge to vector control operation^9^. Resistance defined as the ability in a strain of some organism to tolerate doses of a toxicant that would prove lethal to majority of individuals in a normal population of the same species owing to physiological or behavioural adaptation^12^. Insecticides resistance will often be led to the more frequent use of insecticide, higher dose and can harm the environment. Worldwide, insecticide resistance has been reported. For *Aedes Aegypti*, there is consistently high deltamethrin resistance in Brazil and French Guiana while scattered deltamethrin resistance of *Aedes Aegypti* was found not only in South East Asia, but also in the Middle Eastern region (Saudi Arabia) 9,13. Temephos resistance has been reported in Brazil, French Guiana and the Caribbean whereas lower resistance in some parts of Africa^9^. While resistance to all four groups of insecticide for *Aedes Albopictus* in South East Asia^9^. Particular mechanism of insecticides resistance of the vector has pointed out. This includes reduced penetration of insecticide into the vector, metabolic resistance where there is enhanced enzymatic activity for biodegradation and sequestration of insecticides, a non-synonymous mutation affecting the proteins targeted by insecticides^14^,^15^ and behavioural resistance^12^.

There is no recent published report on insecticide resistance of dengue vector that covers multiple types of insecticide specific to South East Asia. Some studies only report on pyrethroid resistance^16^, and others only study on resistant to insecticide at either larva or adult stage. More studies have done focussing on insecticides resistance for malaria vector compared to *Aedes*. Understanding of the current distribution of insecticide resistance, mechanism of resistance and alternative method to overcome the resistance will allow the implementer of vector control to make an appropriate decision on the appropriate choice of insecticide for the dengue control program. The findings also provide input for conducting surveillance and monitoring of insecticide resistance as well as to evaluate the effectiveness of vector management. Therefore, this systematic review will provide an analysis of the distribution of insecticide resistance of *Aedes* mosquito in South East Asia, to describe the mechanism of insecticide resistance of *Aedes* mosquito in South Asia as well as to identify the possible insecticidresistance management plan.

## Material & Methods

### Search strategy and study selection

A comprehensive search of literature from PubMed, Scopus and ProQuest was performed on the 1^st^ of April 2019 to search for relevant studies. PRISMA checklist used for the workflow of publications search. The text keywords used are “South East Asia” OR Malaysia OR Thailand OR Singapore OR Brunei OR Myanmar OR Cambodia OR Laos OR Philippines OR Indonesia OR “East Timo” OR Vietnam AND Aedes OR dengue OR aegypti OR albopictus AND pesticides OR insecticide OR “dengue control” OR fogging OR organophosphate OR pyrethroid OR larvicide OR temephos OR diphenylmethane AND resistance OR knockdown OR kdr OR mutation with year limit from 2014 to 2019. Articles retrieved from the database and compiled using Mendeley Desktop version 1.19.4 duplicates with 100% matched were removed by the software automatically. Next, the title of each article read by all authors and agreement obtained to exclude articles that did not match with the keywords. If there was any doubt, the abstract was retrieved and read to justify the decision. The abstracts of the articles distributed among the authors for assessment of inclusion and exclusion criteria. Then, selected full articles were retrieved and distributed to the authors. Two independent authors were responsible for examining and extracting the data for each article. At the disagreement point, the third author was consulted.

**Figure 1:**
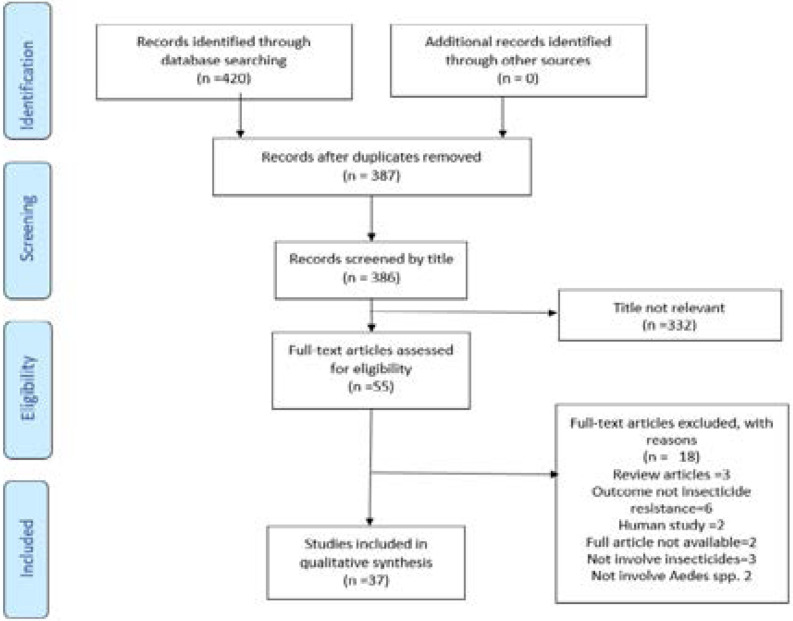
Process of Study Selection

PubMed, Scopus and ProQuest searches identified 420 articles. Thirty-three duplicates were removed. By screening and reviewing for title and abstract, 55 potentially relevant articles were identified and retrieved for more detailed evaluation. Out of these 55 articles, 37 articles fulfilled all the inclusion and exclusion criteria, and subsequently, 18 articles excluded with reasons. There were three articles rejected because of reviewed articles, six articles not related to insecticide resistance, two articles are studies on human, two articles with no full text available, three not involve insecticides, and two articles did not examine on Aedes Aegypti or Aedes Albopictus species. The detail PRISMA flow diagram illustrated in Figure 1.

### Data Extraction and Data Synthesis

Data extraction was done by the authors independently using a predetermined data collection form. The data will then have crossed checked by all authors to minimise errors. Resistance data obtained from all the included articles. In order to find the geographical location of the study sites, the latitude and longitude extracted from the reported article. If the data is not available, the latitude and the longitude of the study site generated using latlong.net (Latlong coordinates reference for World Geodetic System WGS84 standard), based on the study location's name specified in the articles. In this study, the insecticide resistance status categorised based on the latest WHO recommendation^17^, which are more than 98% 24 hours mortality is categorised as “susceptible”, less than 98% 24 hours mortality suggest “resistance”.

The spatial data analysed with QGis software version 3.6. The resistance status was mapped according to the class of insecticide, which are pyrethroid, organophosphate and organochlorine. The data were plotted against the latitude and the longitude to obtain the spatial distribution of insecticide resistance.

## Results

### Descriptive Analysis

Thirty-seven (37) studies selected in this review, published from the year 2015 to 2019. Thirteen (12) articles were from Malaysia, twelve (12) from Indonesia, six (6) from Thailand, one (1) from Laos, one (1) from Vietnam, three (3) from Singapore, one (1) from Cambodia, one (1) includes Asian countries.

The insecticides studied from pyrethroid, organophosphate, organochlorine and carbamate classes. The type of insecticides was varied from one study to another, and with different concentration. The main resistance measurement was a percentage of 24 hours mortality, Knocked Down Time for 50% (KT50) or Knocked Down Time for 95% (KT95), and specific gene mutation. In term of the year study conducted, the earliest year stated in the articles was in 2006. However, not all articles mentioned the year of the resistance studies were conducted. However, the authors estimated the studies should be on average less than years from the publication date^18^. There is the variability of the dosage for specific pesticides tested for the resistance across the study. Most of the studies using the baseline dosage stated by the World Health Organization (WHO)^17^; however, some studies examined the resistance with an escalated dosage of the pesticides. In term of *Aedes* population, adult or larvae or both were tested. Besides, the studies were specific to either *Aedes Albopictus* or *Aedes Aegypti* or both. Table 1 summarises the general information of the included studies.

**Table 1:** Summary of the Characteristics of Included Studies

Subject	Description
Countries	Malaysia (12), Indonesia (12), Thailand (6), Laos (1), Vietnam (1), Singapore (3), Cambodia (1), Asian (1)
Insecticide studied	Pyrethroid, organophosphate, organochlorine, carbamate
Resistance measurement	% mortality (majority), KT_50_ or KT_95_, Gene mutation (16)
Ranges of the year of study	Earliest 2006–2017(estimated)
Dosage comparison	Various, majority studied baseline dosage provided by who or self-determined based on diagnostic dose calculation
Aedes population	Adult or larvae, or both, species-specific

### Mapping the Distribution of Resistance Status

**Figure 2:**
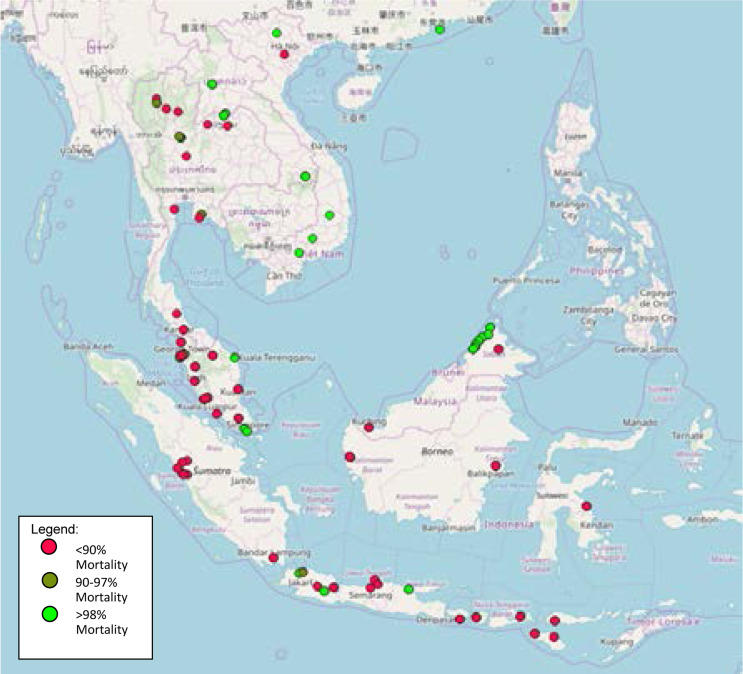
The Distribution of 24 Hours Mortality of Insecticide Resitance, Irrespective of the Class of the Pesticide

Figure 2 illustrates the distribution of overall insecticide resistance irrespective of the class of the pesticides. Majority of the study used discriminating concentrations of the relevant insecticide from WHO bioassay^16^,^17^,^6^, while some were self determinedself-determined based on diagnostic dose calculation. The distribution of the resistance status represented with the percentage of 24 hours mortality of the mosquitoes. The 98% mortality indicates susceptibility, whereby 90% to 97% mortality means the resistance is suggested and need further confirmation. For below 90% mortality, resistance is indicated and did not need further confirmation test.

The insecticide resistance was distributed in a concentrated form at the Peninsular Malaysia and Sumatera Barat region. There is a mixed pattern (susceptibility and resistance) on Java Island and Northern Thailand. Meanwhile, some studies showed susceptibility towards insecticides in West Malaysia, Vietnam and Laos. However, this data was solely on the published data. This means that it displays the presence of the status but did not describe the overall situation of each locality.

**Figure 3:**
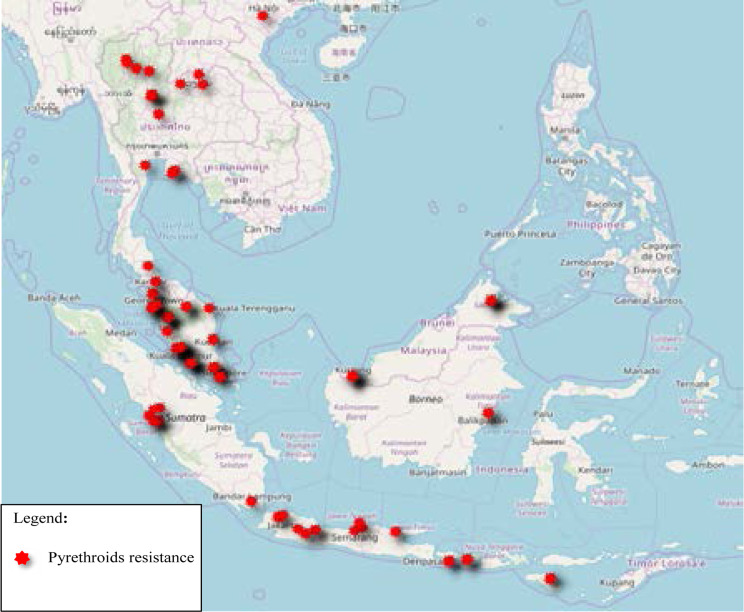
The Distribution of Pyrethroids Resistance Published from 2015 to 2019

**Figure 4:**
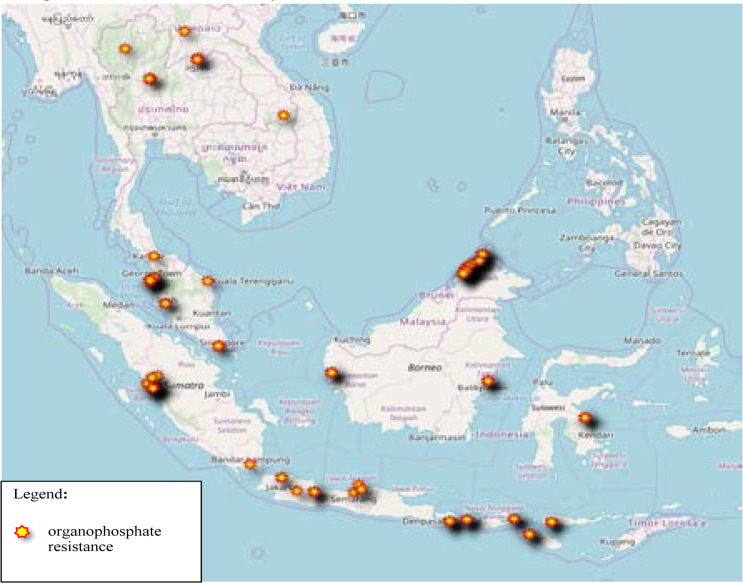
Organophosphate Resistance Based on Published Data from 2015 to 2019

**Figure 5:**
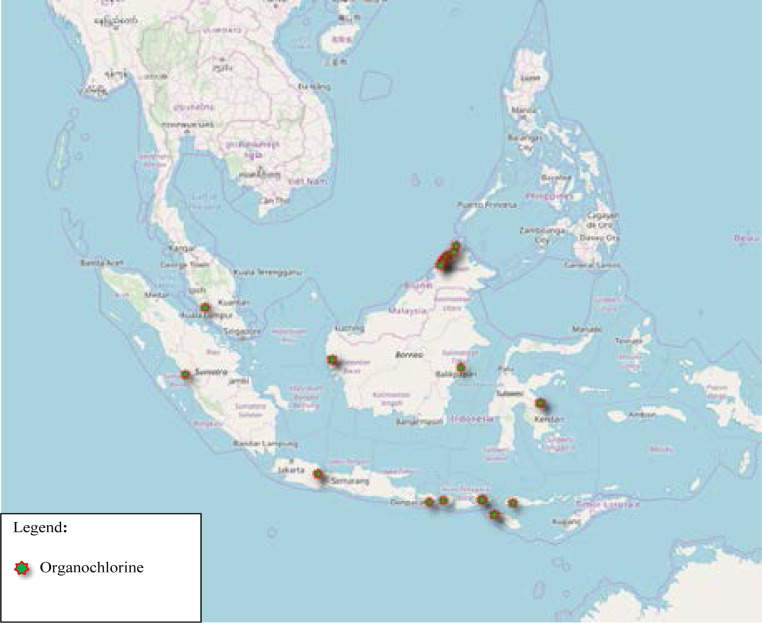
Organochlorine Resistance Based on Published Data from 2015 to 2019

The pyrethroid resistance illustrated in Figure 3 and concentrated on the west coast of Peninsular Malaysia, as well as Northern Thailand. None of the published data on resistance in Vietnam and only one location in Laos. There is scattered distribution throughout Java Island, Indonesia. While for organophosphate, resistance represented by clustered distribution in Sumatera Barat, Indonesia and Sabah, East of Malaysia (Figure 4). Similar pattern as pyrethroid resistance seen across Java Island (Indonesia), for Organophosphate Resistance. Besides, study sites at Sumatera Barat and Northern part of Peninsular Malaysia showed similar resistance to pyrethroid and organophosphate, which suggest the possibility of cross-resistance. Clustered distribution in Sabah, East of Malaysia. Scattered distribution in Nusa Tenggara Barat, Indonesia. However, there is no data on Organochlorine resistance published from Laos, Vietnam and Thailand (Figure 5). Table 2 is the summary of the included studies in this review.

**Table:** 

	Author, year	Location/ strains	Sample	Insecticides	Findings
1	(Ameliayap, Chen, Sofian-Azirun, Lau et al. 2018) 16	Indonesia	*Ae. aegypti* adult	pyrethroid-based mosquito coils containing	KT between d-allethrin and transfluthrin, d-allethrin and metofluthrin, as well as transfluthrin and metofluthrin displayed significant associations, portraying the presence of cross-resistance within pyrethroid insecticides
2	(Boyer et al. 2018)^19^	Cambodia	*Ae. aegypti adult* *and* larvae	Temephos (0.2, 0.05, 0.03, 0.02, 0.01, 0.004 mg/L)	Larvae- lower mortality rate to temephos. Adult- highly resistant to permethrin (pyrethroid)
3	(Chen et al. 2018)^20^	Malaysia	*Aedes albopictus*	Pyrethroid	-All strains of *Ae. albopictus* were most susceptible to metofluthrin, with mortality rates >80%. Mortality rates ranging from 5.0 to 100% were observed from all populations exposed to d-trans allethrin, d-allethrin, and prallethrin
4	(Chin et al. 2017)^21^	Malaysia (11 states)	*Ae. Aegypti*	metofluthrin 0.01% w/w (Fumakilla) d-allethrin 0.20% w/w (Fish A) d-trans allethrin 0.15% w/w (Shieldtox) prallethrin 0.04% w/w (Ridsect)	-Significant associations were detected between the knockdown rates of metofluthrin and d-allethrin, and between metofluthrin and d-trans allethrin, suggesting the occurrence of cross-resistance within pyrethroid insecticides
5	(Elia-Amira et al. 2018)^22^	Malaysia, Sabah	*Ae. albopictus* (Skuse)	Bromophos, malathion (0.125 mg/L) fenthion (0.025 mg/L) fenitrothion (0.02 mg/L) temephos (0.012 mg/L) chlorpyrifos (0.012 mg/L) dichlorodiphenyltrichloroethane (DDT, 0.012 mg/L) dieldrin (0.050 mg/L)	resistant (mortality < 90%) towards malathion, temephos, and DDT. exhibited a wide range of susceptibilities against bromophos, with mortality ranged from 49.33 to 93.33%.
6	(P H Hamid et al. 2018)^23^	Indonesia	*Ae. aegypti*	5% malathion 0.05% deltamethrin 0.75% permethrin 0.05% cyhalothrin 0.1% bendiocarb 0.15% cyfluthrin	Association in V1016G of Aedes with resistance to permethrin but not F1534C
7	(Penny Hum aidah Hamid, Prastowo, Widyasari et al. 2017)^24^	Indonesia	*Ae. aegypti*	5% malathion 0.05% deltamethrin 0.75% permethrin 0.05% λ-cyhalothrin 0.1% bendiocarb 0.15% cyflothrin	Kdr analysis of voltage-gated sodium channel (Vgsc) gene showed significant association to S989P and V1016G mutations linked to resistance phenotypes against 0.75% permethrin. *Ae. aegypti* F1534C gene mutation did not result in any significant correlation to resistance development.
8	(Hasmiwati, Rusjdi & No fita 2018)^25^	Indonesia	*Ae. aegypti* larvae	Temephos pestanal 250 mg 97.5% with 0.02 mg/L	Detection of Ace-1 gene (Genotype variation of T506T location in Ace-I gene)
9	(Hasmiwati, & Supargiyo no 2018)^26^	Indonesia	*Ae. aegypti* larvae	Temephos postanal 250 mg 97.5%, with 0.02 mg/L	populations have point mutations in the VGSC gene corresponding to S989P and V1016G amino acid substitutions. Genes study- to design allele-specific primers to detect the kdr allele mutations.
10	(Haziqah- Rashid et al. 2019)^27^	Indonesia	*Aedes aegypti* larvae	bromophos (0.050 mg/liter) chlopyrifos (0.002 mg/liter) fenitrothion (0.020 mg/liter) fenthion (0.025 mg/liter) malathion (0.125 mg/liter) temephos (0.012 mg/liter) DDT (0.012 mg/liter) dieldrin (0.025 mg/liter)	All field-collected Ae. aegypti larvae were resistant against diagnostic doses of chlorpyrifos, malathion, temephos, and DDT with mortality rates ranging from 0 to 74.67%.
11	(Ishak et al. 2015)^28^	Malaysia	*Aedes* *aegypti* and *Aedes* *albopictus* 2–5-day-old F2	Temephos (1 g/L for larvae 0.75% Permethrin (Type I pyrethroid) 0.05% Deltamethrin (Type II pyrethroid), 4% DDT (organochlorine), 0.1% Bendiocarb (Carbamate) and 5% Malathion (organophosphate) for adult	Knockdown resistance (kdr) Synergist assays with PBO (permethrin, deltamethrin, DDT or bendiocarb) the 24 hours mortality is measured
12	(Ishak et al. 2017)^29^	Malaysia	*Aedes aegypti*	Permethrin 0.75% DDT 4%	% mortality after 24 hours exposure 204 genes were commonly over-expressed with monooxygenase P450 genes (CYP9J27, CYP6CB1, CYP9J26 and CYP9M4) Glutathione S-transferases, carboxylesterases and other gene families commonly associated with insecticide resistance were also over-expressed
13	(Ishak et al. 2016)^30^	Malaysia	*Aedes albopictus*	pyrethroid	Metabolic resistance (cytochrome P450 upregulation) and possibly a reduced penetration mechanism (consistent over-expression of cuticular protein genes) were associated with pyrethroid resistance *CYP6P12* over-expression was strongly associated with pyrethroid resistance *CYP6N3* was over-expressed across carbamate and DDT resistant populations
14	(Kasai et al. 2019)^31^	Asia, Europe and South America	*Aedes albopictus*	permethrin 5.87 ng/female, and 58.7 ng	Genotyping studies detected a knockdown resistance (*kdr*) allele V1016G in *Vssc*. F1534C and F1534S, were also detected
15	(Kongmee et al. 2019)^32^	Thailand	*Ae. Aegypti, 3–5-day-old* *female*	deltamethrin 0.05% +/- piperonylbutoxide (PBO) 4%	Mortality after 24 hours exposure- PBO increase mortality %
16	(Lau et al. 2015)^33^	12 states in Malaysia	F1 larvae of *Ae.* *aegypti and Ae.* *albopictus*	pyriproxyfen 0.5%, Methoprene 1.3%, diflubenzuron 25%, cyromazine 75%, novaluron 10%,	Resistance ratio Insect growth regulators (IGRs; Insecticide for larva stage: - low resistance was detected
17.	(Leong et al. 2018)^34^	Selangor, Malaysia	five – seven days old adult females *Aedes* *Aegypti*	**organochlorine** DDT (98%); **carbamate**: propoxur (99.8%); **organophosphate**: malathion (98.7%), temephos (97.5%); **pyrethroids**: cyfluthrin (99.8%), deltamethrin (99.6%), etofenprox (97.7%), lambdacyhalothrin (97.8%) and permethrin (98.1%)	Synergists ethacrynic acid, S.S.S.- tributylphosphorotrithioate and piperonyl butox ide increased the toxicity of insecticides but failed in certain population Enzyme elevated a-esterase, B - asterase, glutahion, monoxygenase
18	(Low et al. 2015)^35^	Malaysia	*Aedes* *albopictus* (Skuse)	dieldrin	Presence of the A302S mutation
19	(Marcombe et al. 2018)^36^	Lao PDR	F1, F2 larvae of *Aedes* *aegypti*	Bacillus thuringiensis israelensis (Bti), diflubenzuron, pyriproxyfen and spinosad diflubenzuron and temephos	Insecticide susceptibility of *Ae. aegypti* against Bacillus thuringiensis israelensis (Bti), diflubenzuron, pyriproxyfen and spinosad
20.	(Mohiddin et al. 2016)^37^	Penang, Malaysia	*Aedes* *Albopictus* larvae	Temephos 0.012 mg/L (diagnostic dose), 1mg/L (operational dose) and Bacillus thuringiensis subsp. israelensis (Bti) 6000 to 24000 international toxic unit/L	Higher lethal time and resistance ratio were detected from Flat Sri Hamna (a dengue hotspot area
21.	(Mulyanings ih et al. 2017)^38^	Indonesia	*Ae. albopictus* larvae	organophosphates	Resistance status of *Ae. albopictus* larvae to organophosphates
22.	(Pang et al. 2015)^39^	Singapore	*Ae. aegypti.*	delthamethrin-treated net	mortality rate at amino acid residue of alleles V1016G of DIIS6 or F1269C of DIIIS6 was detected in 93% of field strains of Ae.
23.	(Plernsub, et al. 2016) 40	Thailand	*Aedes aegypti*	deltamethrin	Susceptibility of knockdown resistance mutations, S989P, V1016G and F1534C, in a heterozygous genotype to deltamethrin
24.	(Plernsub, S aingamsook, Yanola, Lu mjuan, Tipp awangkosol, Walton, et al. 2016)^41^	Chiang Mai city	*Aedes aegypti*	pyrethroids, deltamethrin	Temporal frequencies of F1534C and V1016G in Ae. aegypti populations The impact of the mutations on the efficacy of thermal fogging with the pyrethroid deltamethrin
25	(Rahim, 2017^42^	Penang, Malaysia	*Aedes Alb* adult	Permethrin Deltamethrin Malathion 2.4% DDT 4%	This study may assist the health authorities to improve future chemical-based vector control operations in dengue-endemic areas
26	Rahim 2016^43^	Malaysia	*Aedes Albopictus* larva	Thermophos Malathion	The revised and established lethal diagnostic dose findings from the current work are crucial to elaborate on the variation in susceptibility of *Ae. albopictus* in future resistance monitoring programs in Malaysia.
27	Rasli 2018^44^	Malaysia	*Aedes Aegypti*	Permethrin (synthetic pyrethroid) deltamethrin and cyfluthrin lambda-cyhalothrin malathion	kdr gene and the detoxification of the oxidase enzyme play a major role in the development of a pyrethroid resistance in *A. aegypti*. **Recommend** Rotational planning of insecticide uses by substituting pyrethroids with organophosphates is highly recommended in localities where *A.* *aegypti* is reportedly highly resistant to pyrethroids but still susceptible to organophosphate. The usage of synergists such as piperonyl butoxide (PBO) could be considered in order to overcome the resistance due to oxidases. Proactive monitoring of the kdr gene throughout all dengue-endemic area in Malaysia is highly suggested as well.
28	Sayono 201 6^45^	Indonesia	*Aedes Aegypti*	Pyrethroid	These findings strongly suggest the need for an appropriate integrated use of insecticides in the region. The 989P, 1016G and 1534C polymorphisms in the *AaNaV* gene are potentially valuable molecular markers for pyrethroid insecticide resistance monitoring.
29	Smith 2017^46^	Singapore	*Aedes aegypti*	Pyrethroid	Two mutations S989P + V1016G, commonly occur together in parts of Asia. These results provide useful information for resistance management and for better understanding pyrethroid interactions with VSSC.
30	Smith 2018^47^	Singapore	*Aedes Aegypti*	Pyrethrod	Genetic Cytochrome P450 monooxygenase (CYP)-mediated detoxification is one of the primary mechanisms of pyrethroid resistance.
31	Sun-on P et al 2018^48^	Chiang ma i, Thailand	Kdr strain -*Aedes* *aegypti* (allele frequency of S989P+V1016G mutation) -DDT, permethrin and deltamethrin	Pyrethroid -0.05% deltamethrin-impregnated papers	-effect of relaxation of deltamethrin selection pressure on the level of mixed-function oxidases (MFO) -aids in the development of new control chemicals, provides information on potential environmental modulators of resistance, and allows for the detection of resistance markers before resistance becomes fixed in the population This study indicates that there was a significant but small reduction in the activity levels of MFOs when pyrethroid selection pressure is relaxed in this kdr strain of *Ae. aegypti*.
32	Susilowati 2018^49^	Tangerang	*Ae. aegypti*	Pyrethroid	Conclusion is *Ae. aegypti* from three districts in Tangerang city have various resistance levels to pyrethroid
33	Tangena 2018^50^	Lao, Vientiane Capital and Luang Prabang	*Aedes albopictus*	DDT, malathion, permethrin, deltamethrin and, temephos	Multiple-insecticide resistance was found. *Aedes albopictus* control efforts in villages need to expand to include secondary forests and rubber plantations, with larval source management and limited use of insecticides.
34	Thanispong K 2015^51^	Thailand Rayong, Koh Chang, and Pong Nom Ron	*Aedes albopictus*	(0.026% deltamethrin, 1.024% permethrin, 0.570% bifenthrin, 0.237% cypermethrin, and 0.035% α-cypermethrin)	Routine assessment of these baseline results should guide future resistance monitoring to pyrethroid insecticides in *Ae. albopictus* in Thailand.
35	Thongwat 2015^52^	Phitsanulok Province, Thailand	*Aedes Aegypti*	temephos, permethrin and deltamethrin	LC50 and mortality rate
36	Widjanarko 2017^53^	Wonosobo subdistrict, Indonesia	*Aedes sp* in	identified that vectors have already developed resistant to organophosphate insecticide, as many as 50% out of the total sample tested	It is important to use another type of insecticide such as pyrethroid.
37	Wuliandri 2015^54^	Yogjakarta, Indonesia	*Aedes Aegypti*	Pyrethroid	-1023G allele is associated with resistance to type I and type II pyrethroids -A resistance advantage conferred to V1023G homozygotes through addition of the S996P allele in the homozygous form

## Discussion

Chemical insecticide use is one of the critical components in the *Aedes* control activity. An insecticide is used to control *Aedes* species through space spraying, insecticide-treated materials, larvicide and residual spraying55. Without proper management, the mosquitoes can develop resistance which can affect the dengue control program. Insecticide resistance had become an essential challenge faced in many dengue-endemic countries, particularly in South East Asia^56^. The World Health Organization (WHO) had recommended the insecticide resistance to be included as a part of integrated entomological surveillance to optimise dengue control activities^17^. Unfortunately, not all resistances information was published by the vector control agencies across the SEA countries. The scarce of published information on the resisance studies causes a comprehensive analysis of the regional threat of insecticide resistance is not possible.

Nevertheless, the reporting trend on insecticide resistance on dengue vectors had enriched since the last 10 years from the last published review by Ranson et al. in 2008^57^. Furthermore, the methodology used were more standardized, based on the WHO recommendation for the later publications. Based on the available data, the mapping of the resistance status showed the abundance of resistance issues in most of the study sites. Insecticide resistance is indeed a burden to the control programs because it will increase the time and the cost for vector management, especially during the outbreak^56^. Furthermore, agricultural sectors will also be affected by the unplanned usage of pesticides in dengue control. Subsequently, the accumulative burden of dengue transmission, agricultural lost and environmental risk may impact the socioeconomic impact of a population and the country.

Besides, the published data also showed there is a possibility of cross-resistance across the study sites, particularly in Indonesia and Malaysia. Cross-resistance occurs when the resistance to one insecticide confers resistance to another type of insecticide^58^. This is due to the probability of sharing a similar mode of action between the insecticides, or the vector develops mechanism, which is resistance to several types of insecticides. For instance, the cross-resistance between Pyrethroid and DDT, which highlighted in the previous studies^59^. As the consequences, this situation will limit the choices of effective insecticide for vector control. Therefore, effective entomological surveillance is necessary as one of the strategies for effective vector control^55^.

## Pyrethroid Resistance

The most common pyrethroid used is Metofluthrin, Permethrin, Deltamerin and d-Allethrin. Over time, the unplanned and frequent use of insecticides has led to the development of resistance against insecticides in mosquitoes. Based on this review, different levels of resistance seemed to the numerous populations despite there is geographical variation in the level of resistance found. This is might due to the intensive use of the chemical with inadequate insecticide management plan^60^. The widespread of the resistance will reduce vector control efficacy and enhanced disease transmission.

The mechanisms of pyrethroid resistance include the mutations in the voltage-sensitive sodium channel gene (target-site resistance) and metabolic-mediated insecticide resistance. The Voltage-sensitive sodium channel (Vssc) gene comprises four homologous domains, and each of them contains six hydrophobic subunits. Mutations occur in the Vssc gene have a linked to knockdown resistance (kdr) in many insect diseases vectors in selected studies. Based on available data, there are three mutations have been detected in Southeast Asia (Indonesia, Thailand, Singapore) which are S989P, V101G and F1534C in *Aedes aegypti* mosquitoes. The detection of these mutations was affirmed to confer sodium channel resistance to pyrethroids, and other associated mutations are still yet to be inspected because the emergence of new kdr is possible when pyrethroid insecticides remained to be the first-line defence in the control of *Aedes Aegypti*.

Metabolic resistance is caused by elevated activity through overexpression or conformational change of enzymes that are involved in the processes of insecticide metabolism, sequestration, and excretion 9. The metabolic enzymes involved belong to large gene families who are cytochrome P450 monooxygenases (P450), glutathione S-transferases (GST), and carboxyl/cholinesterase (CCE). Cytochrome P450 monooxygenase (CYP)-mediated detoxification is one of the primary mechanisms of pyrethroid resistance61. Based on available data, only Malaysia and Singapore reported on Cytochrome P450s (P450s) involved in insecticide resistance reduce the efficacy of insecticide-based vector control by rendering vector control ineffective. The CYP9 and CYP6 family play an essential role in insecticide resistance in *Aedes Aegypti*^28^, ^61^.

## Strategies to Overcome Insecticide Resistance

The insecticide resistance has become a global issue, and there are few strategies to reduce the development of insecticide resistance. The strategies include the insecticides of unrelated classes with different modes of action should be sprayed in rotation, ideally two times per year (WHO 2016). Rotational planning of insecticide uses by substituting pyrethroids with organophosphates is highly recommended in localities where Aedes aegypti is reportedly highly resistant to pyrethroids but still susceptible to organophosphate^44^.

The combination of insecticide with a suitable synergist may help to counter the insecticide resistance and maximise the effectiveness of adulticide operations. The usage of Piperonyl Butoxide (PBO) as synergists could be considered in order to overcome the resistance due to oxidases. For example, the use of Deltamethrin combined with a synergist was effective against resistant populations of Aedes aegypti, the synergistic effect of PBO enhanced knockdown and mortality more rapid by regress the resistance allele and blocked the enzymatic activities that help in detoxification of insecticides^32^. Besides that, the indoor thermal fogging of Deltacide containing S-bioallethrin (a knockdown agent) and Deltamethrin (a killing agent) has a strong adulticidal effect, and these formulated mixtures have shown to be effective against both adult and larval Aedes species^62^.

Public health local agencies may also consider a combination of biological and nonbiological strategies in controlling these vectors. The use of natural enemies in biological control such as the larvivorous fish Poecilia reticulata as a food source for mosquito 63, the entomopathogenic bacteria Bacillus thuringiensis israelensis to destroy the gut lining of mosquito larvae^64^,^65^ and the release of *Aedes aegypti* infected with Wolbachia that cause sterility via cytoplasmic incompatibility 64. In nonbiological, Petroselinum crispum can be used with other chemicals or measures in integrated mosquito management for controlling the vectors, particularly in localities with high levels of pyrethroid resistance 66.

Last but not least, in order to delay or prevent the development of insecticide resistance in vector populations, integrated vector management programs should include a resistance management component 55. Timely, effective entomological assessment and proper data management provided essential information for the management of vector control, and in order to successfully develop and implement of resistance management strategies, knowledge of the mode of action or chemical class of the available insecticide products is crucial^17^.

In Southeast Asia, dengue appears to be endemic. The resistance towards insecticide is geographically varied, which is associated with the practice of insecticide usage. Besides, the existence of cross-resistance also shoud be identified, and the role of genetic mutation and mechanism of resistance are among the strategies to optimise vector control management. Among the management strategies are rotation, combination, integration with synergist and routine assessment of resistance profile. Therefore, surveillance on the insecticide resistance should be conducted according to the best practice recommendations. Lastly, the implementation of successful resistance management strategies against both species is urgently needed. Failure of recognition of this issue will lead to poor control of the disease and will escalate the resources needed to control the vector.
